# Apparent shear stress-based method on an inclined interface plane for predicting discharge in straight compound channels

**DOI:** 10.1016/j.mex.2019.05.027

**Published:** 2019-05-29

**Authors:** Xiaonan Tang

**Affiliations:** Department of Civil Engineering, Xi’an Jiaotong-Liverpool University, Suzhou, 215123, China

**Keywords:** Apparent shear stress-based method for discharge prediction of compound channel flow, Overbank flow, Compound channel, Apparent shear stress, Momentum exchange

## Abstract

Accurately predicting flow discharge in a compound river channel becomes increasingly important for flood risk management and river eco-environment design. This paper proposes a new general approach based on the concept of the apparent shear stress at an inclined interface plane between main channel and floodplains. The new approach with a diagonal plane is applied with a wide range of the author’s experimental data and the data available in the literature, which include 59 datasets. Among them, 27 are homogenous channels of symmetric channels (22 datasets) and asymmetric channels (5 datasets) whereas 32 are heterogeneously roughened channels of symmetric channels (22 datasets) and asymmetric channels (10 datasets). It was found that the new approach improves the accuracy of discharge compared with the DCM for all datasets. The predicted total discharge for straight homogeneous channels has a mean absolute percentage error (MAPE) of 5%, whereas the MAPE error is about 6.7% for heterogeneously roughened channels.

• A general approach of discharge prediction is presented based on apparent shear stress on an inclined interface.
• Both zonal and total discharge can be calculated using the proposed method.
• The predicted results are compared with 59 sets of experimental data along with the DCM.

## Graphical abstract

**Figure:**
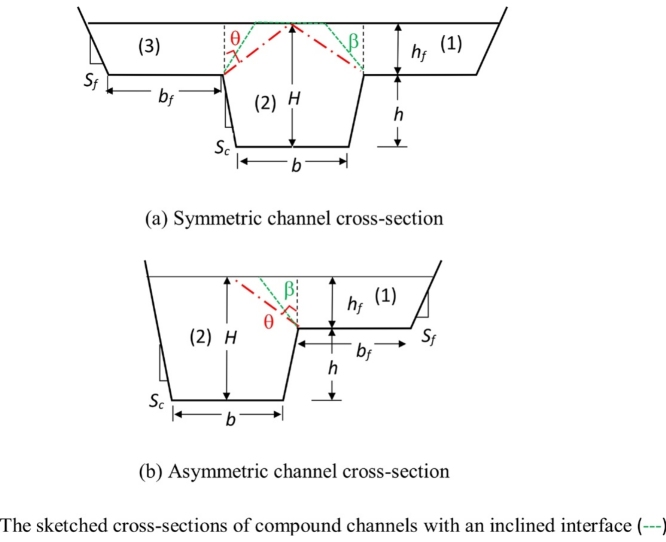

**Specifications Table**

**Table:** 

Subject Area:	• *Engineering* • *Environmental Science*
More specific subject area:	*Compound channel flow*
Method name:	*Apparent shear stress-based method for discharge prediction of compound channel flow*
Name and reference of original method:	*N/A*
Resource availability:	*N/A*

## Method details

**Fig. 1:**
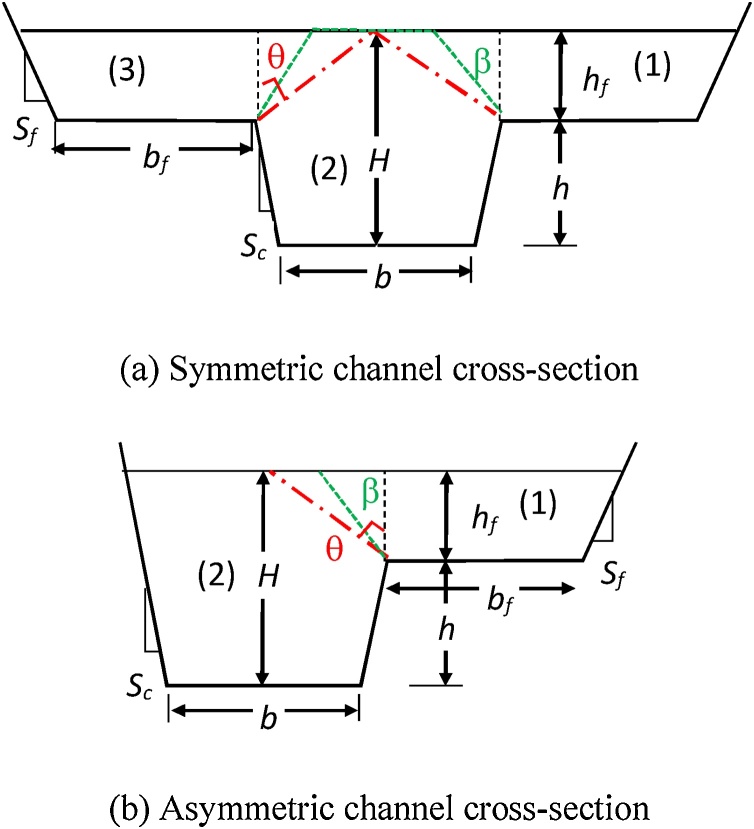
The sketched cross-sections of compound channels with an inclined interface (). (a) Symmetric channel cross-section. (b) Asymmetric channel cross-section.

For the convenience of reference in the subsequent context, the sketched cross-sections of both symmetric and asymmetric compound channels are shown in Fig. 1, where *H*, *h* and *h_f_* are the flow depth of main channel, bankfull, and floodplain (subscript *f*), respectively. *b* and *b_f_* are the widths of the main channel bottom and floodplain, respectively; *S_c_* and *S_f_* are the side slopes of the main channel and floodplain, respectively. The inclined dotted green line is an angel of β with the vertical line that starts from the intersect point between the main channel and floodplain, whereas θ is the angel between the diagonal line (i.e. the dash-dotted red line) and the vertical dash line.

This proposed new method is based on the force balance for each zone, which is separated by an imaginary inclined line as indicated by the green dotted line in Fig. 1, where the apparent shear force on the inclined interface (i.e. the plane along the line of green dotted line at an angle of β) is resulting from the momentum transfer of flow between the main channel and the floodplain. The new method is described as follows:

Based on the streamwise force balance of each part of channels per unit length separated by the vertical line (i.e. the vertical dash line that starts from the intersect point between the main channel and floodplain, see Fig. 1), it follows

(1) $$\rho gA_{c}S_{o}=\rho f_{c}U_{c}^{2}P_{c}+N_{f}\tau_{a}h_{f}$$

(2) $$\rho gA_{f}S_{o}=\rho f_{f}U_{f}^{2}P_{f}-\tau_{a}h_{f}$$

In Eqs. (1) and (2), the left-hand side represents the streamwise component of the gravitational force, and the first term on the right-hand side represents the boundary shear force along the wetted perimeter of the main channel in Eq. (1) and the boundary shear force of floodplain in Eq. (2), respectively. The last term on the right-hand side represents the apparent shear forces along the vertical interface plane between the main channel and floodplains.

In the above equations, *U* is the cross-sectional mean velocity, *A* is the cross-sectional area, *ρ* is the density of fluid, *S_o_* is the bed slope of channel, *h_f_* is the depth of flow at the interface (i.e. the flow depth of floodplain), *P* is the wetted perimeter, *f* is the frictional factor, *N_f_* is the number of floodplain, and the subscripts *c* & *f* denote the main channel and floodplain respectively. The apparent shear stress (*τ_a_*) on the vertical interface plane between main channel and its floodplain can be evaluated by the zonal velocities in each zone, as used by Huthoff et al. [1], Yang et al. [2,3] and Tang [4] and given by

(3) $$\tau_{a}=\frac{1}{2}\rho \alpha_{m}\left(U_{c}^{2}-U_{f}^{2}\right)$$

where α*_m_* is an interface constant.

Similarly when the inclined line at an angle of β is used to separate the channel cross-section to three zones (symmetric channel) or two zones (asymmetric channel), as denoted by zone (1)–(3) in Fig. 1, the apparent shear stress (τ_a_) along the inclined interface is then evaluated by a similar Eq. (3), where the velocity differences are between zone (2) and zone (1) or (3). Take the symmetric compound channel as an example (Fig. 1(a)), the apparent shear stress on each inclined interface is then the same and is given by

(4a) $$\tau_{a12}=\frac{1}{2}\rho \alpha_{12}\left(U_{2}^{2}-U_{1}^{2}\right)$$

(4b) $$\tau_{a23}=\frac{1}{2}\rho \alpha_{23}\left(U_{2}^{2}-U_{3}^{2}\right)$$

where τ_α12_ and τ_α23_ are the apparent shear stress at the inclined interfaces on the left and right, respectively; *α*_12_ and *α*_23_ are the corresponding interface constants due to moment transfer. Because of the symmetry of the channel, τ_α12_ and τ_α23_ are equal since *α*_12_ and *α*_23_ are the same, let them as α_m_. *U* is the average velocity of sub-zone, and subscripts (1–3) denote the sub-zones as shown in Fig. 1(a).

Based on the force balance of each sub-zone (1–3), one can obtain the averaged velocity of each sub-zone. For the symmetric compound channel, take half of the cross-section on the right for analysis (i.e. zones 1 and 2 in Fig. 1(a)). By combining Eq. (3) with Eqs. (1) and (2), it follows that

(5) $$U_{1}^{2}=U_{1,0}^{2}+\frac{\varepsilon_{f}}{1+\varepsilon_{f}+2\varepsilon_{c}}\left(U_{2,0}^{2}-U_{1,0}^{2}\right)$$

(6) $$U_{1}^{2}=U_{1,0}^{2}+\frac{\varepsilon_{f}}{1+\varepsilon_{f}+2\varepsilon_{c}}\left(U_{2,0}^{2}-U_{1,0}^{2}\right)$$

with the coefficients being:

(7) $$\varepsilon_{c}=\frac{1}{2}\alpha_{m}h^{\prime }/f_{c}P_{c}$$

(8) $$\varepsilon_{f}=\frac{1}{2}\alpha_{m}h^{\prime }/f_{f}P_{f}$$

(9a) $$h^{\prime }=h_{f}\sqrt{1+{\left(\text{tan}\beta \right)}^{2}}\;\;\;\text{for β}\leq \text{θ}$$

(9b) $$h^{\prime }=\frac{B_{c}}{2}\;\sqrt{1+{\left(\text{ctan}\;\beta \right)}^{2}}\;\;\;\text{for β}>\text{θ}$$

where *B_c_* is the width of main channel at bankfull, *h_f_* is the depth of flow at the interface (i.e. the flow depth of floodplain), *P* is the wetted perimeter, *f* is the frictional factor, *h*' is the length of the inclined imaginary line, and θ is the angle between the vertical line and a diagonal line (i.e. the dash-dotted line) in Fig. 1. The subscripts 1–3 denote sub-zones, and the subscript (,0) denotes the values based on the divisional channel method without the consideration of apparent shear stress on the same inclined plane, which is exclusive in the calculation of wetted perimeter.

Take a similar analysis as mentioned above, for the asymmetric compound channel (i.e. zones 1 and 2 in Fig. 1(b)), one can obtain that

(10) $$U_{1}^{2}=U_{1,0}^{2}+\frac{\varepsilon_{f}}{1+\varepsilon_{f}+\varepsilon_{c}}\left(U_{2,0}^{2}-U_{1,0}^{2}\right)$$

(11) $$U_{2}^{2}=U_{2,0}^{2}-\frac{\varepsilon_{c}}{1+\varepsilon_{f}+\varepsilon_{c}}\left(U_{2,0}^{2}-U_{1,0}^{2}\right)$$

where the coefficients (ε_c_, ε_f_) are given by Eqs. (7) and (8).

From Eqs. (5), (6), (10) and (11), one can write the following unified equations for *U*_1_ and *U*_2_ in both symmetric and asymmetric compound channels:

(12) $$U_{1}^{2}=U_{1,0}^{2}+\frac{\varepsilon_{f}}{1+\varepsilon_{f}+N_{f}\varepsilon_{c}}\left(U_{2,0}^{2}-U_{1,0}^{2}\right)$$

(13) $$U_{2}^{2}=U_{2,0}^{2}-\frac{N_{f}\varepsilon_{c}}{1+\varepsilon_{f}+N_{f}\varepsilon_{c}}\left(U_{2,0}^{2}-U_{1,0}^{2}\right)$$

where *N*_f_ is the number of floodplain, and the coefficients ε_c_ and ε_f_ are given by Eqs. (7) and (8).

Then based on Eqs. (12) and (13), one can calculate the zonal velocity of both main channel and floodplain as follows:

(14) $$U_{f}=U_{1}$$

(15) $$U_{c}=\left[U_{2}A_{2}+\left(U_{1}-U_{2}\right)N_{f}\;\Delta A_{2}\right]/A_{c}$$

where Eq. (15) is based on the discharge calculation of main channel: *U*_c_ *A*_c_ = *U*_2_ (*A*_2_ – *N*_f_ Δ*A*_2_)  + *N*_f_ *U*_1_ Δ*A*_2_, and Δ*A*_2_ represents the area between the inclined and vertical lines in Fig. 1(a). Note that *A* is the cross-sectional area, and subscripts (c, f) represent the values of main channel and floodplain respectively. Δ*A*_2_ can be calculated by:

(16a) $$\begin{array}{cc} \;\Delta A_{2}=h_{f}^{2}\;\text{tan}\beta /2 & \text{for}\;\;\text{β}\leq \text{θ} \end{array}$$

(16b) $$\begin{array}{cc} \;\Delta A_{2}=\frac{B_{c}}{8}\left(4h_{f}-B_{c}/\text{tan}\beta \right) & \text{for}\;\;\text{β}>\text{θ} \end{array}$$

When β = θ, the inclined imaginary line becomes the diagonal line, the corresponding *U*_c_ in Eq. (15) becomes

(17) $$U_{c}=U_{2}+\left(U_{1}-U_{2}\right)N_{f}h_{f}B_{c}/\left(4A_{c}\right)$$

Eq. (17) is also obtained by Tang [5].

## Detailed procedures of calculation

In summary, to obtain the zonal discharge and total discharge of a compound channel, the following steps are

Step 1: calculate the zonal mean velocity (U_1,0_, U_2,0_)

For given geometry of cross-section like Fig. 1, calculate the zonal mean velocity (U_1,0_, U_2,0_) based on Manning’s formulas (U_0_ = 1/n R ^2/3^ S_o_^½^) using the divisional channel method with the same inclined interface excluded in the calculation of wetted perimeter (i.e. ε_c_ and ε_f_ are zero). n is the Manning's coefficient, R is the hydraulic radius in each zone, and S_o_ is the bed slope.

Step 2: calculate ε_c_ and ε_f_ from Eqs. (7) and (8) with given coefficient α_m_

Step 3: calculate the predicted zonal mean velocity (U_1_, U_2_) based on Eqs. (12) and (13)

Step 4: calculate the zonal velocities of main channel and floodplain, i.e. U_c_ and U_f_, from Eqs. (14) and (15)

Step 5: calculate the zonal discharges and total discharge as follows:

Q_c_ = *U*_c_*A*_c_ = *U*_2_ (*A*_2_ – *N*_f_ Δ*A*_2_)  + *N*_f_*U*_1_ Δ*A*_2_, Q_f_ = *U*_f_*A*_f_

Q_total_ = Q_c_ + N_f_. Q_f_

## Results

Take an example for the case of β = θ, i.e. the diagonal line is taken for the inclined line, the results are given as follows:

A total of 59 datasets of experiments from the literature cover both homogeneous and heterogeneous compound channels, which have various width ratios [channel total width (*B*) at bankfull/main channel bottom (*b*) = 1.5–15.8] and bed slopes (S_o_ = 2.6 × 10^−4^–1.3 × 10^−2^).

**Fig. 2:**
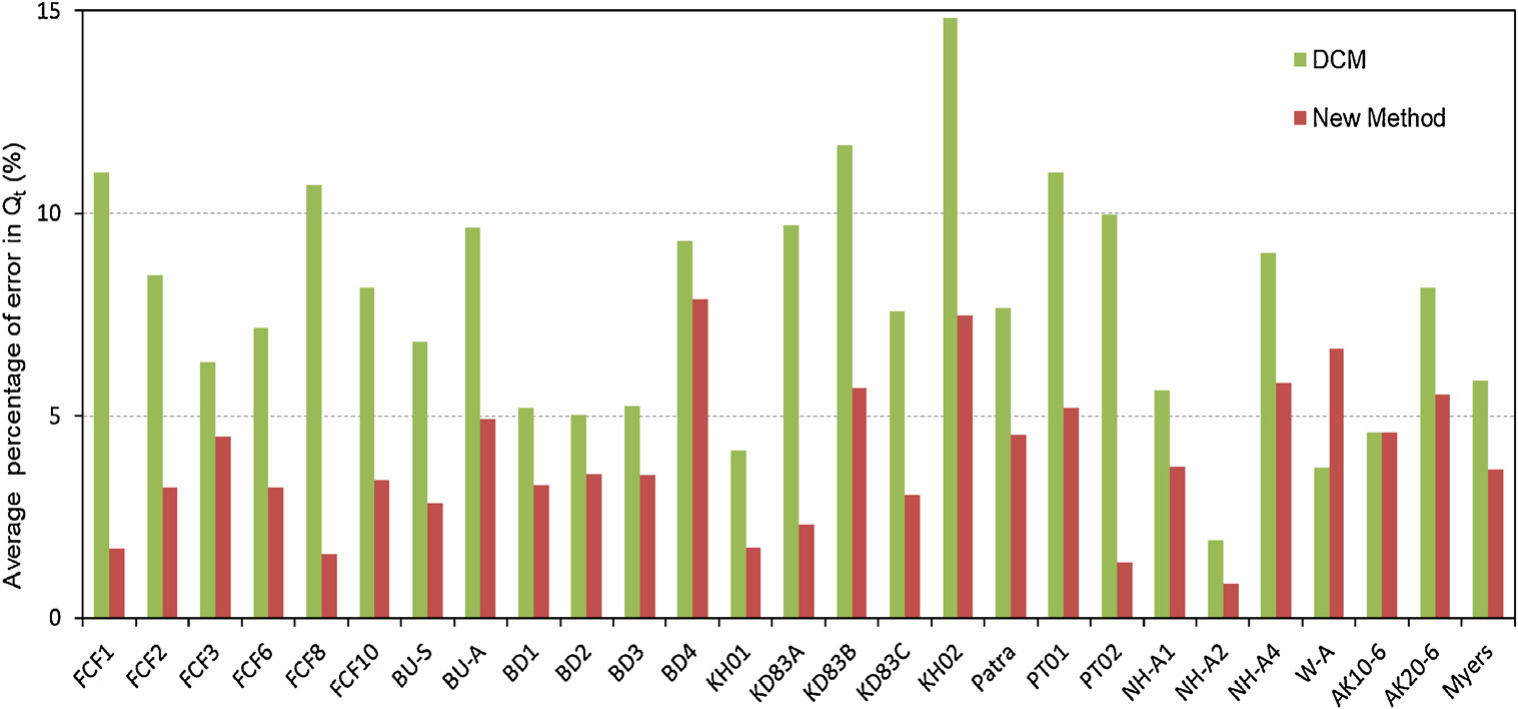
Averaged error percentage of total discharge (%*Q*_t_) for homogenous compound channels.

For homogeneous compound channels, the details of the 27 experimental datasets see Tang [5], which includes the data of a very wide width ratio by Mohanty and Khatua [6]. These data are not listed in this MethodsX paper for the sake of brevity. Fig. 2 shows the comparison results of the present method (termed as new method) and the vertical divisional channel method (DCM) against the 27 datasets of experiments.

**Fig. 3:**
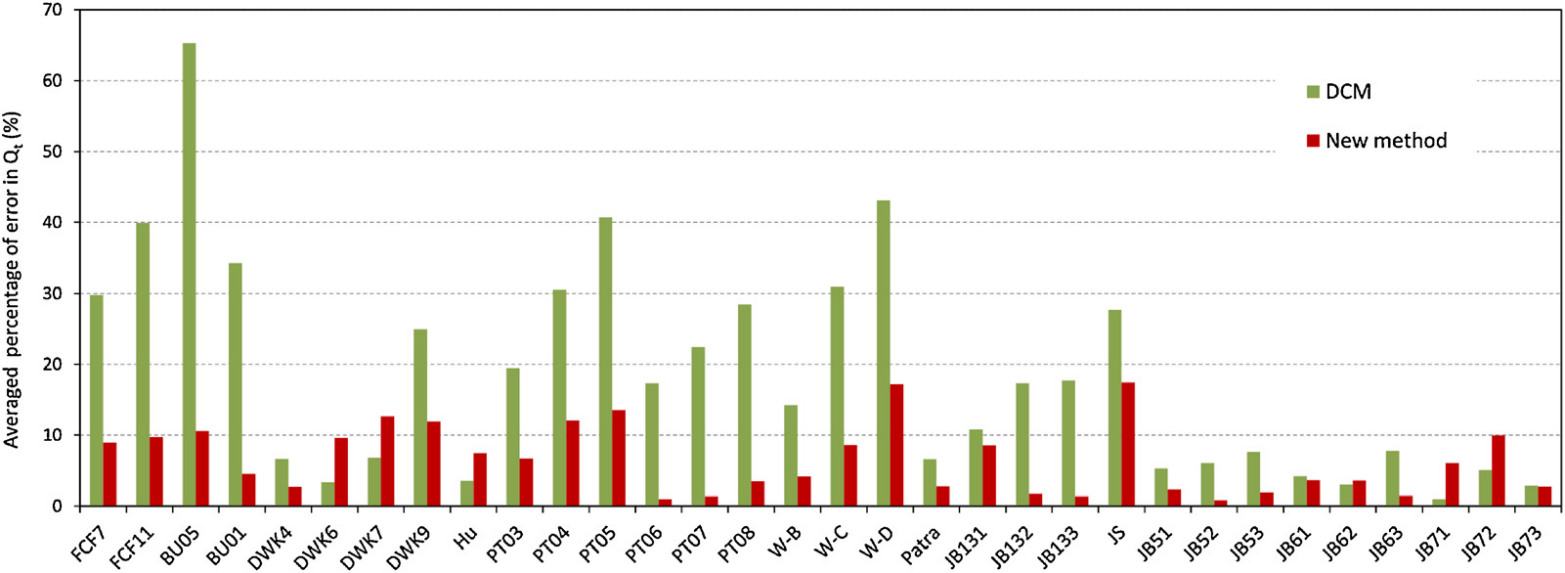
Averaged error percentage of total discharge (%*Q*_t_) for heterogeneously compound channels.

For heterogeneous compound channels, the details of the 32 experimental datasets see Tang [7]. Fig. 3 shows the comparison results of the present method and the DCM with the 27 datasets of experiments.

Note that DCM in Fig. 2, Fig. 3 is the conventional divisional channel method with the vertical line excluded, the new method is based on the inclined interface with β = θ, and the optimal values of α_m_ are founded to be 0.001 and 0.02 for homogenous and heterogeneous compound channels flows respectively in this study.

The mean absolute percentage error (MAPE) of predicted discharge was used as a criterion for the precision of method [8,9]. The percentage of error in predicted discharge of each flow depth is calculated by,

(18) $$\%E_{Q,i}=\frac{\left|Q_{cal,i}-Q_{exp,i}\right|}{Q_{exp,i}}\times 100\%$$

where %*E_Q,i_* is the error percentage of predicted discharge, and *Q_cal,i_* and *Q_exp,i_* are the predicted and observed discharge at *i*th flow depth, respectively. Therefore, the average error percentage of each method for an experiment can be obtained by

(19) $$\%E_{Q}=\frac{1}{N}\sum_{i=1}^{N}(\%E_{Q,i})$$

where *N* is the total number of runs in an experiment.

## Conclusion

Based on the proposed method, the predictive discharge shows significant improvement compared with the DCM for both homogeneous and heterogeneous compound channels. In the future, the proposed optimal values of α_m_ need to be further investigated when more data are available.
